# The Psychological Effects of Short-Term Fasting in Healthy Women

**DOI:** 10.3389/fnut.2016.00027

**Published:** 2016-08-22

**Authors:** Ellen Watkins, Lucy Serpell

**Affiliations:** ^1^East London and City Foundation NHS Trust, London, UK; ^2^University College London, London, UK; ^3^North East London Foundation NHS Trust, Dagenham, Essex, UK

**Keywords:** healthy individuals, fasting, starvation, psychology, emotions

## Abstract

**Objective:**

The study aimed to investigate affective responses to 18-h fasting in healthy controls. In particular, the study focused on self-reported mood, irritability, sense of achievement, reward, pride, and control.

**Method:**

Participants were a non-clinical sample of 52 women with a mean age of 25. A repeated-measures design was used, whereby participants provided diary measures of psychological variables throughout both 18-h fasting and non-fasting periods.

**Results:**

Fasting led to increased irritability, and also to positive affective experiences of increased sense of achievement, reward, pride, and control.

**Discussion:**

Even short-term fasting in healthy controls can lead to positive psychological experiences. This lends support to cognitive-behavioral and cognitive-interpersonal models of ANR, which suggest that dietary restriction is maintained through positive reinforcement.

## Introduction

By definition, individuals with anorexia nervosa (AN) severely restrict their food intake, which can be seen as a mixture of both short-term food deprivation and chronic malnourishment (1, 2). While short-term fasting in healthy individuals has significant differences from the chronic calorie deprivation seen in AN, fasting in healthy participants can offer some clues to the cognitive and emotional changes in AN (3, 4). There is limited research into the psychological effects of starvation in healthy individuals, however, existing research has mainly focused on negative affective and cognitive changes, such as increased anxiety and irritability and poorer concentration (5–7), and increased rigidity and obsessionality (5). Interestingly, studies have indicated that unintentional weight loss can trigger AN (8, 9). This points toward the possibility that starvation itself may have an impact on the development or maintenance of AN (10).

Several models of the development and treatment of AN inherently assume that dietary restriction leads to positive affective experiences. The cognitive-behavioral model posits that AN is maintained, in part, by positive reinforcement from successful restriction [for example, Ref. (11–14)]. Other models, for example, Schmidt and Treasure (15) cognitive-interpersonal approach, also incorporate positive experiences as a result of dietary restriction as important maintaining factors in AN. However, no studies in healthy individuals have as yet focused on the possible positive emotional consequences of fasting in experimentally induced starvation.

### The Current Study and Hypotheses

The study aimed to investigate individual responses to short-term fasting. The study aimed to determine whether fasting would lead to increases in measures of positive mood, reward, hunger, irritability, achievement, pride, and self-control. Due to the scarcity of literature on the psychological consequences of fasting, no directional hypotheses were made.

## Materials and Methods

### Participants

Participants were 52 female volunteers with a mean age of 25.52 years (range 18–56). Participants were eligible if they were healthy, female, aged over 18, and spoke fluent English. Exclusion criteria included current diagnosis of an eating disorder (ED), pregnancy, or knowingly having any medical condition that would make fasting dangerous, such as diabetes. The largest ethnic group in the sample was Chinese (*N* = 15, 28.8%), followed by White Other (*N* = 11, 21.1%), White British (*N* = 8, 15.4%), and Other (*N* = 7, 13.5%). All participants were educated to at least GCSE level, and most were current students (*N* = 31, 59.6%) or employed (*N* = 7, 13.5%). Participants were paid £15 or, if they were an undergraduate student, had the option of receiving course credits for their time.

### Sample Size

A power analysis for this study was carried out based on estimates of effect size from ED research comparing a clinical sample with healthy controls (16, 17). It was estimated that a sample size of 34, with an alpha level of 0.05 would provide 80% power to detect a medium effect size.

### Procedure

The study took place at University College London (UCL) from November 2011 to June 2013. Ethical approval was granted by the UCL Research Ethics Committee. To minimize the risks of fasting, participants were given advice and information on fasting and its possible risks, and were informed to immediately stop fasting if they felt unwell.

### Study Design

The study used a within subjects, repeated-measures design to test individuals’ responses to 18-h fasting and non-fasting periods. Participants met with researchers on three occasions; the first meeting consisted of a screening questionnaire and completion of ED and mood measures. The second and third meetings followed the fasting and non-fasting conditions.

Participants were required to independently complete both an 18-h fasting and an 18-h non-fasting period, which were at least a week apart to ensure no carry over effects of fasting. During the fasting period, participants were asked not to consume any food or drink other than water. Participants were asked to be truthful about whether they had adhered to fasting conditions. In order to promote adherence, participants were informed that a random sample of participants would be asked to provide a urine sample to check levels of ketones, biological markers of fasting. No urine samples were actually obtained as ketone levels are not an accurate indicator of fasting adherence due to biological homeostatic mechanisms (3, 18). During the non-fasting period, participants were told to eat and drink normally.

**Figure 1:**
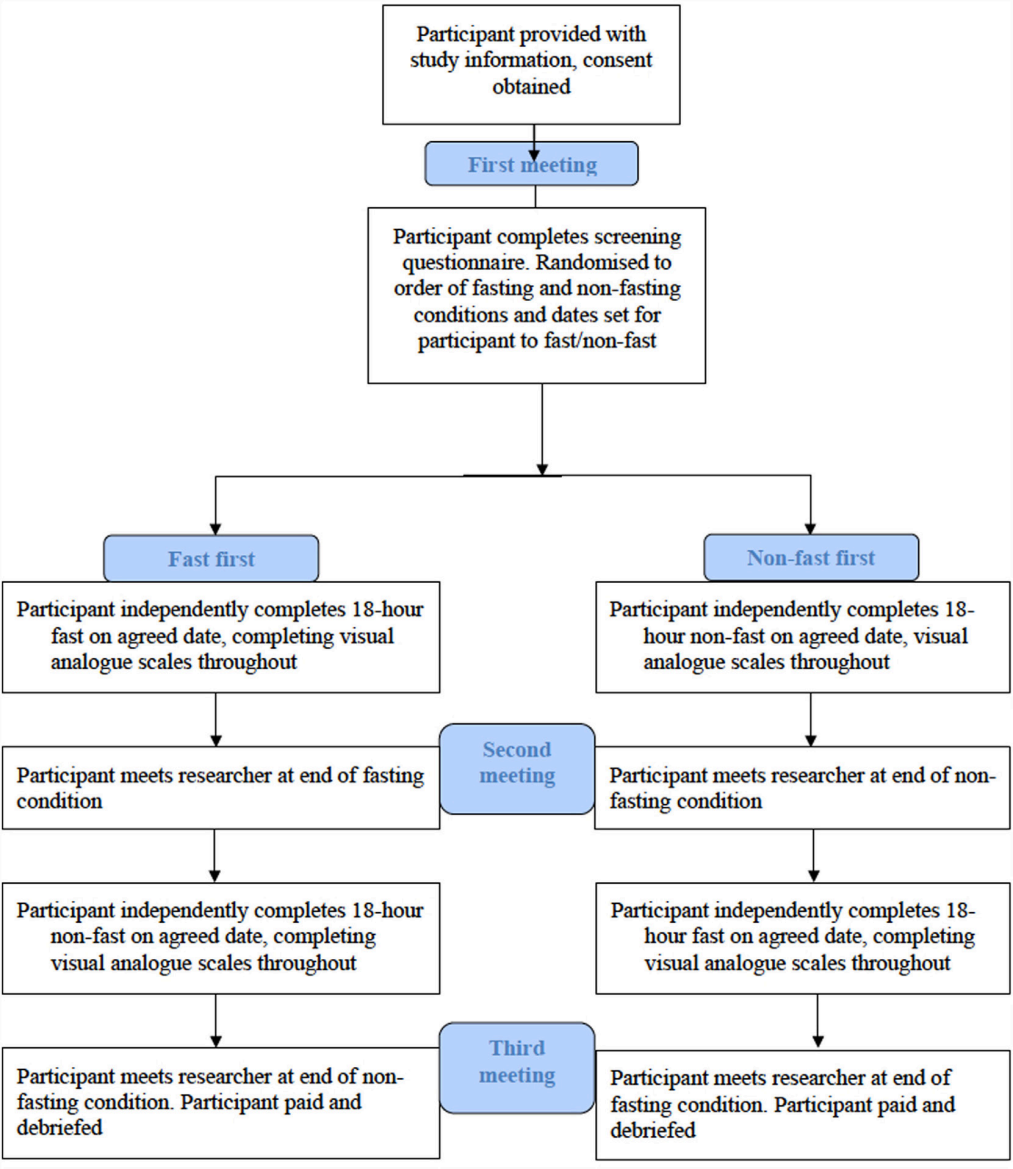
**Flow chart of the study design**.

Participants completed visual analog scales (VASs) diary measures throughout both fasting and non-fasting periods. At the end of both conditions, participants met with researchers to provide their completed self-report measures and, at the end of the third meeting, participants were reimbursed and debriefed. Figure 1 shows a flow chart of the study process.

### Measures

#### Screening Questionnaire

At the initial meeting, participants completed a screening questionnaire, which included basic demographic questions about current physical and mental health, and history of EDs. Participants were also asked the number of meals they usually ate in a day, whether they had fasted before, and how they anticipated they would find fasting. Additionally, participants were asked to rate their mood on a VAS ranging from 0 (“not at all happy”) to 10 (“happiest I could be”).

#### Self-Reported ED Pathology

To screen for ED symptomatology, the Eating Disorder Examination Questionnaire [EDE-Q6; (19)] was used. This is a 28-item self-report questionnaire, providing four subscale scores; dietary restraint, shape concern, weight concern, and eating concern, as well as a global score. A study indicated that the EDE-Q6 has adequate reliability and validity in community samples (20).

#### Self-Reported Depression and Anxiety

Participants completed the Hospital Anxiety and Depression Scale [HADS; (21)], which is a widely used 14-item self-report measure with seven anxiety and seven depression items. It provides separate subscale scores for anxiety and depression, as well as an overall score.

#### VAS Diary Measures

During the fasting condition, participants were asked to rate mood, reward from fasting, sense of achievement, sense of self-control, and difficulty fasting, using VASs ranging from zero (“not at all”) to 10 (“extremely”). Measures were completed every 2 h throughout the fasting day, giving a total of five measures. Participants also provided VAS scores at the beginning of the fasting period to give a baseline. Similarly, throughout the non-fasting condition, participants were also asked to rate mood, irritability, sense of self-control, and sense of achievement so that participants acted as their own controls by providing data on a non-fasting day.

### Randomization

Participants were randomized to complete either the fasting or non-fasting condition first. For allocation to condition, a computer-generated list of random numbers was used, participants of which were assigned to in the order that they entered the study.

### Statistical Analyses

Data were analyzed using SPSS 19.0 for Windows. All statistical tests used a 0.5 significance level. All participants’ data were analyzed together, and there were no outliers or data excluded. Effect of fasting was assessed using paired samples *t*-tests comparing VAS data before and after fasting. A repeated-measures ANOVA was used to assess whether there was a main effect of fasting.

## Results

### Previous Fasting

Most participants had not fasted before (73.1%, *N* = 38). Fourteen participants had fasted previously (26.92%). Of these, nine had done so for dietary or health reasons, and five had done so for religion.

### Self-Reported Eating Disorders

Mean BMI was 21.19 (SD = 3.22, range 15.80–30.70). Eight participants had a BMI below the World Health Organisation (22) clinical cut-off of <18.5 kg/m^2^. Of these, none scored in the clinical range on any EDE-Q subscales.

Mean scores on all EDE-Q subscales were higher than in Fairburn and Beglin (19) normative sample. Mean scores on global, restraint, shape concern, and weight concern subscales were within one SD of Fairburn and Beglin’s norms. Mean eating concern score was within two SDs of the norm.

### Self-Reported Depression and Anxiety

The mean anxiety score was 5.57 (SD = 4.17, range 0–18) and mean depression score was 2.82 (SD = 2.83, range 0–12). These are within one SD of norms found in non-clinical populations previously (23).

### Effect of Fasting

Paired samples *t*-tests were used to compare mean scores on VAS measures at the start of fasting (^F1^) and end of fasting (^F6^). Significance values were Bonferroni corrected to control risk of type-I error. All paired samples *t*-tests can be seen in Table 1. At the end of fasting, participants were significantly hungrier, more irritable, had a significantly higher sense of achievement, pride, and self-control than at the start of fasting. Participants also rated fasting as increasingly difficult throughout the fasting period. Difficulty rating at the end of the fasting period (^F6^) was significantly correlated with hunger, *r* = 0.67, *p* > 0.001, and irritability, *r* = 0.68, *p* > 0.001, at the end of fasting.

**Table 1:** **Paired samples *t*-tests comparing mean scores on VAS measures from start of fasting (^F1^) to end of fasting (^F6^), start of non-fasting (^NF1^) to end of non-fasting (^NF6^) and comparing scores on VAS measures at the end of fasting (^F6^) and end of non-fasting (^NF6^)**.

	Paired samples *t*-tests of VAS scores from start of fasting ^F1^ and end of fasting ^F6^	Paired samples *t*-tests of VAS scores from start of non-fasting ^NF1^ and end of non-fasting ^NF6^	Paired samples *t*-tests of VAS scores from end of fasting ^F6^ and end of non-fasting ^NF6^
Mood	*t*(47) = 2.10, *p* = 0.41	*t*(45) = 0.08, *p* = 0.94	*t*(43) = 0.85, *p* = 0.39
Sense of reward	*t*(47) = −0.66, *p* = 0.51	*t*(45) = 1.40, *p* = 0.39	*t*(43) = −3.17, *p* = 0.03*
Hunger	*t*(47) = −9.05, *p* <0.01**	*t*(45) = −1.44, *p* = 0.16	*t*(43) = −6.99, *p* <0.05**
Irritability	*t*(47) = −3.95, *p* <0.01**	*t*(45) = −1.19, *p* = 0.24	*t*(43) = −2.40, *p* = 0.15
Sense of achievement	*t*(47) = −7.26, *p* <0.01**	*t*(45) = −1.08, *p* = 0.29	*t*(43) = −3.83, *p* <0.01**
Sense of pride	*t*(47) = −6.43, *p* <0.01**	*t*(45) = 0.19, *p* = 0.85	*t*(43) = −4.08, *p* <0.01**
Sense of control	*t*(47) = −4.20, *p* <0.01**	*t*(45) = 0.06, *p* = 0.95	*t*(43) = −1.59, *p* = 0.12
Difficulty	*t*(46) = −6.87, *p* = 0.00**	/	/

Means, SDs, and ranges are calculated based on participants who provided data on respective variables. Significance levels on t-tests are Bonferroni corrected. *indicates p < 0.05. **indicates p < 0.01. Significance values were Bonferroni adjusted.

By contrast, paired samples *t*-tests showed that there was no significant difference on any VAS measures from the start of the non-fasting period (^NF1^) to the end of non-fasting (^NF6^).

*T*-tests were also performed comparing mean scores on VAS measures from the end of fasting ^F6^ and end of non-fasting ^NF6^ periods. These showed that at the end of fasting compared to the end of non-fasting participants experienced a significantly higher sense of reward, were significantly more hungry, had a significantly higher sense of achievement, and a significantly higher sense of pride.

A repeated-measures ANOVA revealed that there was a significant main effect of fasting on VAS measures, *F*(1, 41) = 46.92, *p* < 0.05. There was a significant interaction effect between fasting condition and VAS measure rating, *F*(4.31, 176.56) = 12.09, *p* = < 0.05, suggesting that fasting impacted on participants’ subjective psychological experiences.

## Discussion

The current study set out to explore the impact of short-term fasting on psychological experiences, including positive moods in healthy participants. The hypothesis that fasting would affect psychological variables was supported. As fasting progressed, participants reported that it was increasingly difficult and they became hungrier, however, they also experienced an increased sense of achievement, pride, and control. Although sense of reward did not increase significantly from start to end of fasting, this may be due to the fact that, even at the very start of fasting, participants reported having a higher sense of reward. Sense of reward did continue to increase throughout fasting, though not significantly. At the start of non-fasting, sense of reward was rated as lower and this decreased throughout non-fasting, leading to a significantly higher sense of reward at the end of fasting than at the end of non-fasting.

Previous research has suggested that fasting results in biological symptoms of depression, such as irritability, mood lability, and low libido (24, 25). The results of the current study support these findings, as participants reported feeling increasingly irritable throughout fasting but did not report significant changes in overall mood.

However, as well as negative emotional correlates, this study also found that positive affective experiences, such as a sense of achievement, were associated with the fasting process. While care must be taken in extrapolating from short-term fasting to AN, these findings are at least consistent with cognitive-behavioral or cognitive-interpersonal models of AN, which postulate that AN is maintained partly by positive reinforcement from successful dietary restriction [for example, Ref. (11–15)]. These authors suggest that food restriction is partly reinforced by a variety of resultant positive feelings, such as a sense of control, power, satisfaction, or pride. Previous research indicates that sufferers of AN experience both pros and cons of the disorder. Pros of AN may include making sufferers feel special through their ability to maintain dietary restriction (26). To our knowledge, this is the first study showing that positive feelings can be generated even from short-term fasting in healthy controls. These findings may go some way to elucidating how extreme dietary restriction in AN may begin in vulnerable individuals from even short-term fasting or normal dieting leading to increases in positive mood states. The results may lend support to cognitive-behavioral or cognitive-interpersonal treatment models of AN, which often incorporate exploration of the factors that positively reinforce dietary restriction, and the pro-anorectic beliefs the individual holds.

### Limitations of the Study

The study relied on self-report data, which is potentially more fallible than data from objective assessments or semi-structured interviews due to social desirability bias, distortion, or participants misunderstanding questions. Furthermore, data from the fasting and non-fasting periods was completed independently by participants. Although we asked participants not to retrospectively complete missed data points, they may have done so, and we had no way of checking this. However, many participants missed data at one time point (often first thing in the morning), suggesting that, on the whole, they did not retrospectively complete measures, leaving them blank instead.

Data from the fasting and non-fasting periods were obtained using VASs as it was not possible to repeatedly use standardized measures of constructs, such as low mood, which often refer to longer time periods, such as 2 weeks. The VASs provided a simple, fast, repeatable way of obtaining data without overwhelming participants. However, there were shortcomings to gathering data in this way, such as participants having different conceptualizations of affective labels, and positioning of marks not necessarily being comparable across individuals. This was overcome to some extent with the repeated-measures design, which meant that participants acted as their own controls. However, it may be beneficial to repeat this research with use of more standardized measures.

Researchers did not objectively assess whether participants had EDs, other than by using self-reported ED status and the EDE-Q as a screening tool. Future studies should use a full clinical screen to ensure that no participant meets criteria for an ED. Additionally, researchers had no way of determining whether or not participants had adhered to fasting conditions. However, the difference in VAS measures between fasting and non-fasting suggests that participants did fast. Researchers and participants were aware of the condition; therefore, responses on VAS measures could have been biased by participants’ expectations of the effects of fasting. It was not possible in this brief exploratory study to obtain self-report data on dietary intake on non-fasting days, nor on physical activity. Future studies should include these data.

The study was exploratory in nature. Due to this, and having multiple hypotheses, multiple statistical tests were used, thereby increasing the risk of type-I error. Corrections were performed to account for this, however, it would be of benefit to repeat this research with a larger sample to allow for more rigorous statistical testing.

Generalizability of the results to people with EDs is limited by the fact that the sample was non-clinical and mostly comprised students. It would be of benefit to replicate the research with a clinical sample, although this may raise ethical concerns, in light of research on the effects of starvation.

The largest ethnic group in the sample was Chinese, which may have led to errors in completing questionnaires, although all participants described themselves as fluent English speakers. Furthermore, it may also have resulted in participants having lower overall body weight (27). However, all participants in the study were living and studying in the U.K.

The study may have attracted individuals who liked fasting, or experienced ED pathology. This would bias the results, particularly if participants regularly fasted for religious reasons and associated it with positive effects. However, most participants reported that they had never fasted. Moreover, fasting was rated as increasingly difficult over time, indicating that even those who had fasted before found it difficult. In addition, participants may have been attracted by the monetary reward rather than an interest in EDs or fasting.

### Strengths of the Study

Several strengths of the study must also be acknowledged, including the within-subjects design whereby participants acted as their own controls. Additionally, asking participants to provide VAS measures of psychological variables two-hourly throughout the fasting period meant that we obtained rich data. To the best of our knowledge, this is one of the only studies to explore the effect of short-term fasting on psychological variables in healthy controls.

## Conclusion

The results showed that fasting led not only to negative affective states, such as irritability, but also to positive psychological experiences, such as increased sense of reward, achievement, pride, and control as participants reported increasing hunger and difficulty of fasting. To our knowledge, this is one of the only studies showing that these positive psychological experiences can result from fasting, even in healthy controls. These findings lend support to cognitive-behavioral and cognitive-interpersonal models of AN, which suggest that fasting is maintained partly through positive reinforcement, and may elucidate how extreme dietary restriction can result from ordinary dieting or fasting.

## Ethics Statement

This study was carried out in accordance with the recommendations of the Ethics Committee at University College London with written informed consent from all participants. All participants gave written informed consent in accordance with the Declaration of Helsinki.

## Author Contributions

LS and EW conceived of the study and developed the hypotheses. EW drafted the manuscript and LS made substantial contributions to the writing process.

## Conflict of Interest Statement

The authors declare that the research was conducted in the absence of any commercial or financial relationships that could be construed as a potential conflict of interest.
